# Concurrent Dengue and Malaria in Cayenne Hospital, French Guiana

**DOI:** 10.3201/eid1504.080891

**Published:** 2009-04

**Authors:** Bernard Carme, Severine Matheus, Gerd Donutil, Olivia Raulin, Mathieu Nacher, Jacques Morvan

**Affiliations:** Centre Hospitalier de Cayenne, Cayenne, French Guiana (B. Carme, G. Donutil, O. Raulin, M. Nacher); Institut Pasteur de la Guyane, Cayenne (S. Matheus, J. Morvan); Université Antilles – Guyane, Cayenne (B. Carme, M. Nacher); Institut National de la Santé et de la Recherche Médicale (Inserm), Cayenne (B. Carme, M. Nacher)

**Keywords:** Malaria, Plasmodium vivax, Plasmodium falciparum, dengue, co-infection, French Guiana, dispatch

## Abstract

Dengue–malaria co-infection reports are scarce. Of 1,723 consecutive febrile patients in Cayenne Hospital, 238 had dengue (174 early dengue fever cases) and 393 had malaria (371 acute malaria); 17 had both. Diagnosis of 1 of these 2 infections should not rule out testing for the other infection.

Despite a wide overlap between malaria- and dengue-endemic areas, published reports of co-infections are scarce in the literature. The first 2 patients with concurrent malaria (*Plasmodium falciparum*) and dengue were identified in July 2005 (*1*) and November 2006 (*P. vivax*) (*2*). Since then, a few publications described proven or suspected associations, but always as isolated cases (*3**–**6*).

In French Guiana, a French territory in South America that is 92% covered by Amazon rain forest, malaria and dengue fever represent 2 major public health concerns. The annual number of *P. falciparum* and *P. vivax* malaria cases ranges from 3,500 to 4,500. In addition, all 4 dengue virus serotypes have been isolated in the country (*7*). To determine the frequency of concurrent infection with dengue and malaria in French Guiana, we conducted a 1-year study of patients evaluated in the emergency department of Cayenne Hospital.

## The Study

We carried out a retrospective study by testing blood and serum samples on 1,740 patients who consulted the emergency department of Cayenne Hospital seeking treatment for fever compatible with malaria and/or dengue during a 1-year period, July 2004–June 2005 (Figure). Diagnosis of malaria has always been quick; dengue diagnosis was initially conducted only in malaria-negative patients. In our study, dengue investigations were conducted retrospectively at the Pasteur Institute of French Guiana for 99% of patients (1,723/1,740) by using serum samples obtained at admission and frozen at –80°C. Medical records of case-patients with dengue–malaria co-infections were consulted retrospectively to look for severe or abnormal features.

**Figure:**
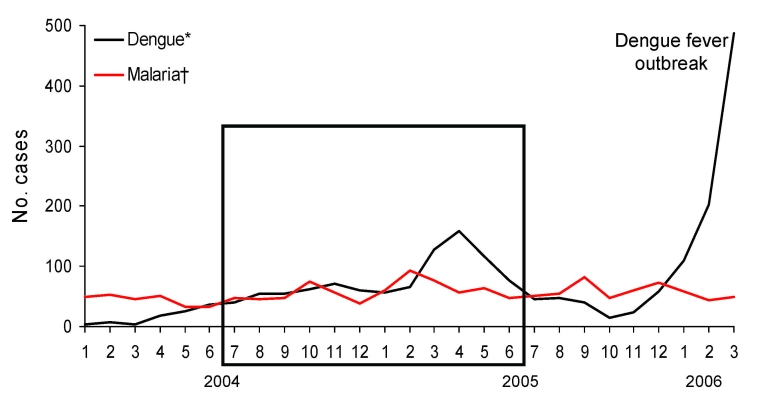
Comparison of confirmed cases of dengue fever and of symptomatic malaria in patients examined at the emergency department of Cayenne Hospital, Cayenne, French Guiana, January 2004–March 2006. The black frame corresponds to the period of the retrospective study (July 2004–June 2005). *Cases confirmed by positive test results from reverse transcription–PCR or virus isolation (Pasteur Institute, Cayenne). †Cases diagnosed based on recorded fever or history of fever in the previous 24 h associated with microscopic detection of asexual forms of *Plasmodium* spp. by blood smear.

Malaria diagnosis was based on the identification of hematozoa on a thin blood film and/or on a thick blood film stained with Giemsa. The screening sensitivity was ≈6 plasmodia/µL (1/1,000 leukocytes). The asexual parasite load (PL) was quantified in percent parasitized erythrocytes for values >0.1 %. For lower values, classes were created using thick smears: class 1, <0.00125% but positive; class 2, >0.00125% but <0.0125%; and class 3, >0.0125% but <0.125%. Asymptomatic *Plasmodium* spp. carriage was considered for classes 1 and 2 (in the absence of prior antimalarial treatment and for case-patients residing >1 year in an area of malaria transmission). Virus isolation or reverse transcription–PCR (RT-PCR) according to Lanciotti et al. (*8*) was performed on all serum samples obtained during the acute phase of infection, between day 0 and day 4 (n = 264). For malaria-positive samples, virus isolation was conducted on all samples without a date of onset of disease (n = 163).

Serologic immunoglobulin (Ig) M testing was performed on all serum samples (n = 1,723). Dengue was detected in 238 case-patients (13.8%); among these, 73% (174/238) were confirmed by positive virologic diagnosis (isolation or RT-PCR), whereas 27% were probable dengue cases (positive IgM serology only). The first group was named early dengue cases (EDC) and the second group late dengue cases (LDC).

Of the 1,723 patients, 393 (22.8%) had smear-positive malaria; of those, 251 (63.9%) were *P.* *vivax,* 133 (33.8%) were *P.* *falciparum*, 2 were *P. malariae*, and 7 were mixed *P.* *vivax* and *P.* *falciparum*. Most malaria-positive case-patients had a parasite count above class 2 (371/393 [94.4 %]), indicating acute malaria.

Concurrent dengue (EDC and LDC) and malaria were confirmed in 17 of the 1,723 patients (1%), corresponding to 7.1% (17/238) of dengue cases and 4.1% (16/393) of malaria cases, respectively (Table). When considering acute malaria associated with EDC, the percentages of confirmed associations were 3.4% for dengue (6/174, 95% confidence interval [CI] 0.7–6.2) and 1.6% for malaria (6/371, 95% CI 0.3–2.9). All 17 associations were considered clinically as malaria, including the 2 case-patients with low parasite counts. Antimalarial drugs were administered promptly in every case. Dengue serology and virology reports were available after the initial episode; however, these results did not influence patient management. Among the 6 acute concurrent infections, none was severe. The clinical evolution was always favorable. Three patients were hospitalized, all in the IgM-seropositive group, i.e., LDC; only 1 was severely ill. This patient, who had *P. vivax* malaria infection, was hospitalized for interstitial pneumonia with severe anemia. Intubation, blood transfusion, and antimicrobial drugs were required, but he was discharged from the intensive care unit after 11 days. No causative agent was identified for this pneumonia. The second patient was hospitalized for diabetes, the third because treatment with Riamet (arthemether + lumefantrine) was only available to inpatients.

**Table:** Clinical course and diagnosis in 17 case-patients with confirmed or suspected concurrent dengue and malaria, Cayenne Hospital, Cayenne, French Guiana, July 2004–June 2005*

Patient no./birth year	Malaria diagnosis				Hospitalized†	Initial diagnosis and clinical signs	Dengue diagnosis			Conclusion
	Species	Parasitemia					IgM	RT-PCR	Iso	
		%	Class							
1/1983	*Pv*	<0.1	C3		No	Malaria	–	+ DEN-1	ND	Confirmed acute concurrent disease
2/1984	*Pv*	0.15	C4		No	Malaria, Tp 51,000	–	+ DEN-3	ND	Confirmed acute concurrent disease
3/1973	*Pv*	0.8	C4		No	Malaria	–	+ DEN-3	ND	Confirmed acute concurrent disease
4/1975	*Pf*	0.1	C3		No	Malaria	–	+ DEN-3	ND	Confirmed acute concurrent disease
5/1983	*Pv*	0.1	C3		No	Malaria	–	+ DEN-3	ND	Confirmed acute concurrent disease
6/1980	*Pv*	0.2	C4		No	Malaria, Tp 52,000, Hb 9.4	SeroC	–	ND	Confirmed acute concurrent disease
7/1981	*Pf*	<0.1	C1		No	Malaria‡	–	ND	DEN-3	Confirmed concurrent infection
8/1953	*Pv*	<0.1	C3		Yes§	Malaria, Tp 71,000	+	–	–	Suspected concurrent infection
9/1952	*Pv*	0.8	C4		No	Malaria, Tp 30,000, BP 80/50	+	–	–	Suspected concurrent infection
10/1948	*Pv*	0.3	C4		No	Malaria, Tp 70,000	+	–	–	Suspected concurrent infection
11/1982	*Pv*	0.25	C4		No	Malaria	+	–	–	Suspected concurrent infection
12/1979	*Pf*	0.4	C4		Yes¶	Malaria	+	–	–	Suspected concurrent infection
13/1956	*Pv*	0.5	C4		Yes#	Malaria, Tp 63,000, Hb 5.9, Sat 83%	+	–	–	Suspected concurrent infection
14/1976	*Pv*	0.7	C4		No	Malaria	+	–	–	Suspected concurrent infection
15/1988	*Pv*	0.5	C4		No	Malaria, Tp 29,000, BP 90/50	+	–	–	Suspected concurrent infection
16/1974	*Pv*	0.1	C3		No	Malaria, HR 146 bpm	+	–	–	Suspected concurrent infection
17/1961	*Pv*	<0.1	C2		No	Malaria‡	+	–	–	Suspected concurrent infection

*IgM, immunoglobulin M; RT-PCR, reverse transcription–PCR; Iso, isolate; *Pv*, *Plasmodium vivax*; – , negative; + positive; DEN, dengue; ND, not done; *Pf*, *P. falciparum*; Tp, thrombocytopenia++ (<100,000 platelets/µL); Hb, hemoglobin (reference range <10 g/L); BP, arterial blood pressure (systolic/diastolic); Sat, blood oxygen saturation; HR, heart rate; bpm, beats per minute. †Hospitalization >8 hours. ‡Possible asymptomatic carrier of *Plasmodium* spp. §For diabetes requiring insulin. ¶For treatment with Riamet (arthemether + lumefantrine). #For interstitial pneumonia.

## Conclusions

Malaria and dengue must be suspected in febrile patients living in or returning from areas endemic for these infections. Although the usual places of contamination by malaria and dengue viruses are quite different in French Guiana, considering that the incubation phase is longer for malaria than for dengue and that the population’s mobility is high, a simultaneous clinical expression of the 2 diseases is plausible. Moreover, in French Guiana, dengue viruses have spread to malaria-endemic rural areas (*9*).

The confirmation of malaria is rapid, and after malaria is confirmed, dengue is usually ruled out without screening for it. Two methods can confirm dengue: dengue-specific IgM seroconversion or detection of dengue virus particles during the acute phase (day 0 to day 4 after onset of fever) by RT-PCR, which is faster and more specific. In published case reports (*1*–*7*), the diagnosis of dengue infection is usually made based on positive dengue IgM; however, this cannot confirm recent dengue, because IgM can persist for months and cross-react with other arboviruses (*10*). If RT-PCR requires a specific laboratory and cannot be performed on site, a new test, the Platelia, is now easily included in any laboratory and is indicated particularly for early-acute phase samples (*11*). To investigate the frequency of dengue and malaria co-infection, the Platelia test should be used in all cases of dengue-like or malaria-like syndrome, even when malaria diagnosis was positive, in regions where both infections may overlap.

Of the 1,723 patients investigated in this study, 17 had concurrent dengue and malaria. In 10 of these patients, recent acute dengue fever could not be confirmed (LDC). Two patients, 1 of whom was part of the EDC group, could have been asymptomatic carriers of *Plasmodium* spp. (1 patient with *P. falciparum* and 1 with *P. vivax*) because of low parasitemia (*12*). A true acute concurrent infection (strictly defined diagnosis) was confirmed in 6 case-patients. Concurrent acute malaria and recent dengue fever had a lower frequency than predicted by the multiplication of both prevalences, but such reasoning implies the same overlapping contamination areas for malaria and dengue, which it is not the situation in French Guiana. The greater prevalence of LDC than EDC associated with acute malaria infection illustrates the prolonged persistence of specific IgM or IgM cross-reaction, which increases the probability of a malaria case when comparing the short 4–5 day period corresponding to EDC. Virologic investigations using isolation or RT-PCR techniques were not performed on samples taken after the 4th day because of the usual disappearance of viremia. Additional associations where fever was initially caused by malaria and followed by dengue after the 4th day of malaria fever could have been undiagnosed.

EDC were diagnosed on average after 4 days of fever, never 5. Thus, delayed complications of dengue or malaria may not be detected using this definition. Such complications could be observed in patients considered LDC. One of these patients had pneumonia, which has recently been described as a complication of *P. vivax* (*13*).

Although acute concurrent infections were benign in our study, special attention should be given to the possibility of co-infection with malaria and dengue, especially when *P. falciparum* is implicated. The distinction between severe dengue and severe malaria must be made in an emergency department or hospital setting because in both situations, early diagnosis is essential for patient care.
